# Facial growth in patients with unilateral cleft lip and palate at 19 years of age after three different one-stage palatal repairs: a longitudinal study with prediction from cephalograms at 5 years of age

**DOI:** 10.1093/ejo/cjae066

**Published:** 2025-03-19

**Authors:** Petra Peterson, Konstantinos Parikakis, Agneta Karsten

**Affiliations:** Department of Molecular Medicine and Surgery, Karolinska Institutet, SE-171 77 Stockholm, Sweden; Department of Plastic Surgery and Craniofacial Surgery, Karolinska University Hospital, SE-171 76 Stockholm, Sweden; Division of Orthodontics and Pedodontics, Department of Dental Medicine, Karolinska Institutet, Box 4064, SE-141 04 Huddinge, Sweden; Division of Orthodontics and Pedodontics, Department of Dental Medicine, Karolinska Institutet, Box 4064, SE-141 04 Huddinge, Sweden

**Keywords:** cephalometrics, long-term outcome, UCLP

## Abstract

**Objectives:**

To compare cephalometric long-term outcomes in patients with unilateral cleft lip and palate (UCLP) and treated with three different surgical protocols for palatal repair. Furthermore, to investigate growth longitudinally and evaluate the possibility to predict the outcome at age 19 from cephalometric values at 5 years.

**Materials/Methods:**

Lateral cephalograms of 68 patients, operated according to the Veau–Wardill–Kilner technique (*n* = 13), the minimal incision technique (*n* = 39), or MIT with muscle reconstruction (MITmr) (*n* = 16) were assessed. At a mean age of 19.0 (SD 0.7) years, 17 skeletal and 6 soft tissue variables were analysed using analysis of variance (ANOVA) with pairwise comparison. Lateral cephalograms at a mean age of 5.1 (SD 0.4) years, from 32 of the 68 patients were used to predict values at 19 years, using a multiple linear regression.

**Results:**

There were statistically significant differences between the three surgical techniques for eight of the skeletal variables and for two of the soft-tissue variables at 19 years. The angle between the sella/nasion plane and the nasion/A plane (SNA) was 74.5 (SD 3.8) after Veau-Wardill-Kilner (VWK), 77.6 (SD 5.3) after minimal incision technique (MIT), and 76.7 (SD 2.6) after MITmr. Adjusted for baseline values, at 5 years, only face height had a significant effect dependent on surgical technique.

**Limitations:**

Due to the exclusion criteria or missing medical records, only 43% of 157 consecutive patients could be included in the study.

**Conclusion:**

MIT and MITmr resulted in better cephalometric results regarding facial growth sagittally and vertically compared to VWK. Most of the cephalometric variables measured showed a strong positive relation between the value at 5 and the value at 19 years of age.

## Introduction

Assessing mid-facial growth after treatment of unilateral cleft lip and palate (UCLP) is essential and should be performed at the end of skeletal maturation to establish if there are factors that could reduce the degree of growth restriction. Cephalometric analysis is an important tool used by most centres performing cleft surgery to analyse long-term outcome of growth and development. Today, there is no controversy regarding the fact that cleft treatment results in some degree of maxillary retrusion [1, 2]. In a systematic review and meta-analysis by Choi *et al.* [3] the recurrence of orthognathic intervention after treatment of UCLP was 30%. However, whether the type and/or the timing of primary palatal repair influences long-term mid-facial hypoplasia is still debated [4–6]. It is challenging to compare different surgical protocols and to divide them into one- or two-stage palatal repairs becomes misleading since both timing, sequence (soft or hard palate first) and technique of palate repair may differ. This could explain why reports of seemingly similar procedures can produce contradictory results [7, 8] or conversely, different techniques seem to result in similar outcomes [9]. Mueller *et al.* [10] stated that it might not necessarily be that the surgical trauma per se compromises growth zones. Maybe, it is in what exact location the surgical trauma is located and probably also at what time point. However, some authors argue that factors such as caseload and experience of the surgeon might explain the variation in outcome to the same extent as the treatment protocol itself [11] and that burden of care, in terms of number of interventions, should be equally important when judging the efficacy of cleft care [12]. The analysis of longitudinal data should also strive to identify ways of predicting, as early as possible, if new treatment protocols seem to result in better, or worse, long-term outcomes.

There are still few studies presenting cephalometric results in adults with UCLP and more information on how to find early predictive variables for successful long-term results after primary palatal surgery is imperative.

The aim of the present research was to investigate and compare the outcome of facial growth at 19 years of age, studied with cephalometric analysis, in individuals with UCLP and treated with three different one-stage techniques for palatal repair. A secondary aim was to investigate the possibility to predict long-term maxillary growth outcome from age 5 to finished growth.

## Research questions

1) Is there a difference in facial growth assessed with cephalometry in young adults after three different types of one-stage palatal repair?
2) Is it possible to predict long-term facial growth in patients with UCLP at 19 years of age from their cephalometric values at 5 years of age?

## Null hypotheses

1) There is no difference in facial growth between patients operated with three different types of one-stage palatal repair.
2) There is no relation between the facial growth at 5 and 19 years of age.

## Material and methods

### Treatment protocols

From 1975 to 1986, children born with UCLP in Stockholm, Sweden were treated in hospital with presurgical maxillofacial orthopaedics, using an external device (T-traction), from the first weeks of life. At the average age of 5 months (SD 1.5) cheiloplasty according to Tennison-Randall and primary rhinoplasty according to McComb was performed together with early bone-grafting to the alveolar cleft. Palatoplasty according to Veau–Wardill–Kilner (VWK) was then performed at a mean of 19 months (SD 2.6). In 1987, T-traction was abandoned in favour of a less invasive method for preoperative orthodontic treatment with a palatal plate and, at the same time, the technique for palatal repair was changed into the ‘minimal incision technique’ (MIT) at the average age of 13 months (SD 3.1). Bone grafting was also postponed until the mixed dentition (mean age 10, SD 1.5). Without changing the timing (13 months, SD 1.1), but with the intent to improve speech outcome, the method for palatal repair was refined in 1998 with the addition of a radical muscle reconstruction (MITmr) according to Sommerlad [13, 14]. The preoperative palatal plate was in this period used when the tongue was placed within the cleft. The techniques and timing for lip- and nose repair were unaltered throughout the whole study period. The palatal surgery evaluated was performed by five high-volume surgeons, as defined by Bearn *et al.* [15].

### Study population

**Figure 1.**
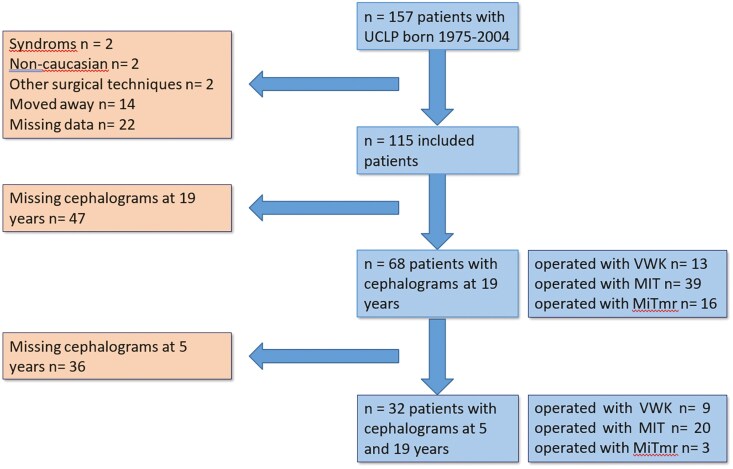
Description of the exclusion process leading to the final study material. Sixty-eight patients, out of 157 born with UCLP between 1975 and 2004, were selected. Thirty-two of the 68 patients also had a lateral cephalogram at the age of 5 years.

The medical records of 157 consecutive patients born from 1975 to 2004 with a complete UCLP (including Simonart’s band of less than three millimetres) and treated by the Stockholm Craniofacial Team were examined. Eighty-nine patients had to be excluded (Fig. 1), leaving 68 patients with lateral cephalograms at 19 years of age to be evaluated.

### Method

**Figure 2.**
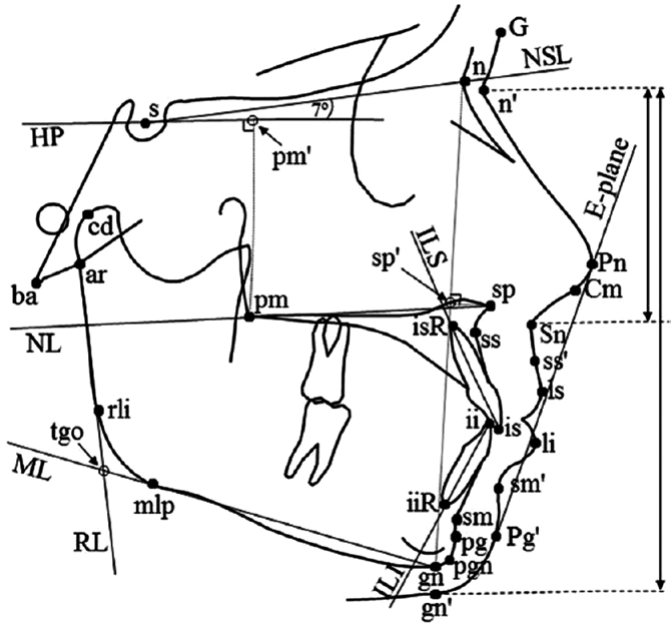
Reference points, lines, and measurements used in the study. ar (articulare): a mid-planed point located at the intersection of the posterior border of the ramus with the inferior surface of the cranial base; ba (basion): the most inferior point on the anterior margin of foramen magnum; cd (condylion): the mid-point on the contour of the glenoid fossa where the line indicating maximum mandibular length intercepts the contour of the fossa; gn (gnathion): the most inferior point on the bony chin; ii (lower incisor tip): the tip of the crown of the lower central incisor; iiR (lower incisor apex): the root apex of the lower central incisor; is (upper incisor tip): the tip of the crown of the upper central incisor; isR (upper incisor apex): the root apex of the upper central incisor; mlp (inferior gonion): a mid-planed point at a tangent to the inferior border of the mandible near gonion; n (nasion): junction of the frontal and nasal bones at the naso-frontal suture; pg (pogonion): the most anterior point on the bony chin; pgn (prognathion): the point on the contour of the bony chin indicating maximum mandibular length measured from the temporomandibular joint; pm (pterygomaxillare): the posterior limit of the floor of the nose at the tip of the posterior nasal spine; pm’: constructed point at the perpendicular projection of pm on HP; rli (posterior gonion): a mid-planed point at a tangent to the posterior border of the ramus near gonion; s (sella): the mid-point of the sella turcica; sm (supramentale): the deepest point in the concavity of the anterior mandible between the alveolar crest and pogonion; sp (spinal point): the anterior limit of the floor of the nose at the tip of the anterior nasal spine; sp´: constructed point at the perpendicular projection of sp on n–gn line; ss (subspinale): the deepest point in the concavity of the anterior maxilla between the anterior nasal spine and the alveolar crest; tgo: constructed point at the intersection between RL and ML; Cm (columella): a landmark on the inferior surface of the nose, representing the anterior delimiter of the naso-labial angle; G (glabella): the most anterior point on the forehead, in the region of the supra-orbital ridges; gn’ (soft tissue gnathion): the most inferior point on the soft tissue chin, in the region inferior to gn; li (labrare inferius): the muco-cutaneous border of the lower lip; ls (labrare superius): the muco-cutaneous junction of the upper lip and philtrum; n’ (soft tissue nasion): the deepest point in the soft tissue concavity overlying the naso-frontal suture; Pg´ (soft-tissue pogonion): the most anterior point on the soft-tissue chin; Pn (pronasale): the most anterior point on the nasal tip; sm’: the deepest point in the concavity between li and the soft tissue chin; Sn (subnasale): the junction of the columella of the nose with the philtrum of the upper lip; ss’ (superior labial sulcus): the deepest point in the concavity of the upper lip, midway between Sn and ls. E-plane (esthetic plane): Pn-Pg’; HP (constructed Horizontal Plane): a line −7° from NSL passing through s; ILI (Line of lower incisors): ii-iiR; ILS (line of upper incisors): is-isR; ML (mandibular line): gn–mlp; NL (nasal line): sp–pm; NSL (nasion sella line): n–s; RL (ramus line): ar–rli. Hard-tissue variables: SNA° (s–n–ss), SNB° (s–n–sm), ANB° (ss–n–sm), NAPg° (n-ss-pg), NSL/NL°, NSL/ML°, NL/ML°, gonial angle° (ML/RL), palatal plane length mm (sp–pm), mandibular length mm (cd–pgn), n–sp´/n–gn (%), sp’-gn/n-gn (%), posterior upper Face Height mm (pm–pm´), posterior Face Height mm (s–tgo), ILS-NL°, ILI-ML°, ILI-ILS°. Soft-tissue variables: soft ANB° (ss’-n’-sm’), facial convexity° (G–Sn–Pg´), nasolabial angle° (Cm-Sn-ls), NSnNGn (%): n’-Sn/n’-gn’(vertical distance, see double arrows), upper lip to E plane mm (ls from E-plane), lower lip to E plane mm (li from E-plane).

Analogue lateral cephalograms of the UCLP group were scanned (Epson Perfection V700 Photo, Seiko Epson Corp., Japan) at 300 dpi and an 8 bit grey scale to produce digitized cephalograms. Magnification was adjusted to 0 for all radiographs. Tracing was performed with a cephalometric program (Viewbox 4.0.1.7, dHAL Software, Kifissia, Greece) as a computerized tracing technique is equally reliable to hand tracing but less time consuming [16]. All available lateral cephalograms, 68 taken at 19 years of age and 32 taken of the same patients at 5 years, were traced blindly and randomly twice by one of the authors (Costas Parikakis), an orthodontist who is not a member of the cleft-team, and the mean values of the two digitizations were used. Reference points defined by Solow and Tallgren [17] and Legan and Burstone [18] were used (Fig. 2). Measurements based on landmarks difficult to identify were excluded for every individual film. Tools for image manipulation, available by the cephalometric program, were used before the decision to omit specific points. The intra-observer errors of measurements were estimated through a random selection of 15 cephalograms. The same 15 cephalograms were retraced after a two-week interval. The observer method errors si were calculated using the Dahlberg’s formula [19].

The three groups of patients operated according to VWK, MIT, and MITmr techniques, respectively, were analysed and compared with analysis of variance (ANOVA). To reveal the relation between values at 5 and 19 years, a multiple linear regression model was performed. The model was adjusted for surgical technique. Confidence intervals were calculated at a 95% level. The cephalometric data were compared to pooled mean values, at 19 years of age, for both genders of individuals born without a cleft from the study by Thilander *et al.* [20], where normal values for males and females were presented separately. Independent *t*-test was used to compare all three surgical techniques to normative data. The statistics were performed with the software R version 4.1.2 [21].

### Ethical approval

The study was approved by the ethical committee of Stockholm nr 2008/502-31/2.

## Results

### Differences between the three surgical techniques at 19 years of age

**Figure 3.**
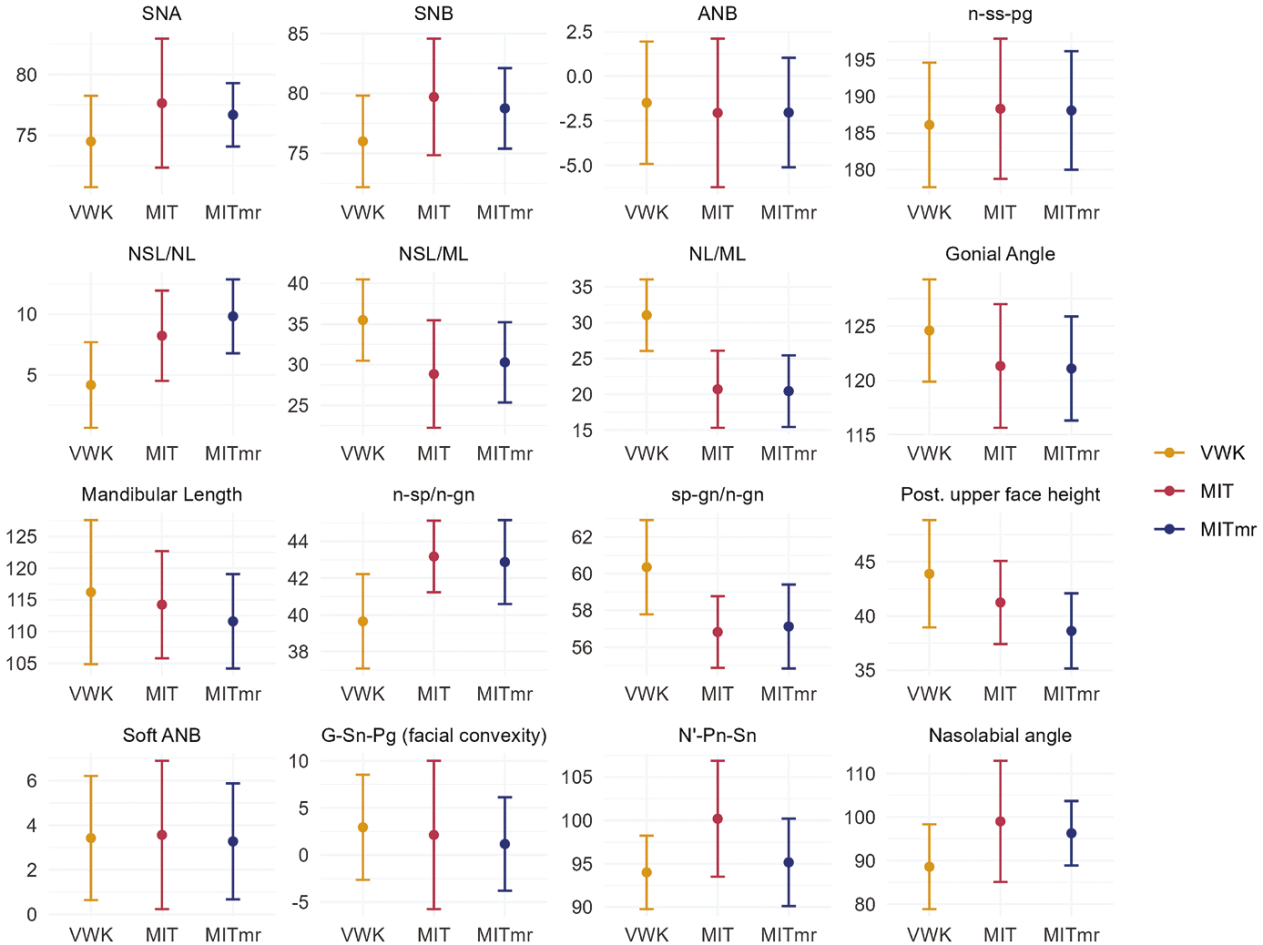
The average value with +/− one standard deviation at 19 years of age, divided into groups of surgical techniques (VWK, MIT, MITmr) (*n* = 68).

**Figure 4.**
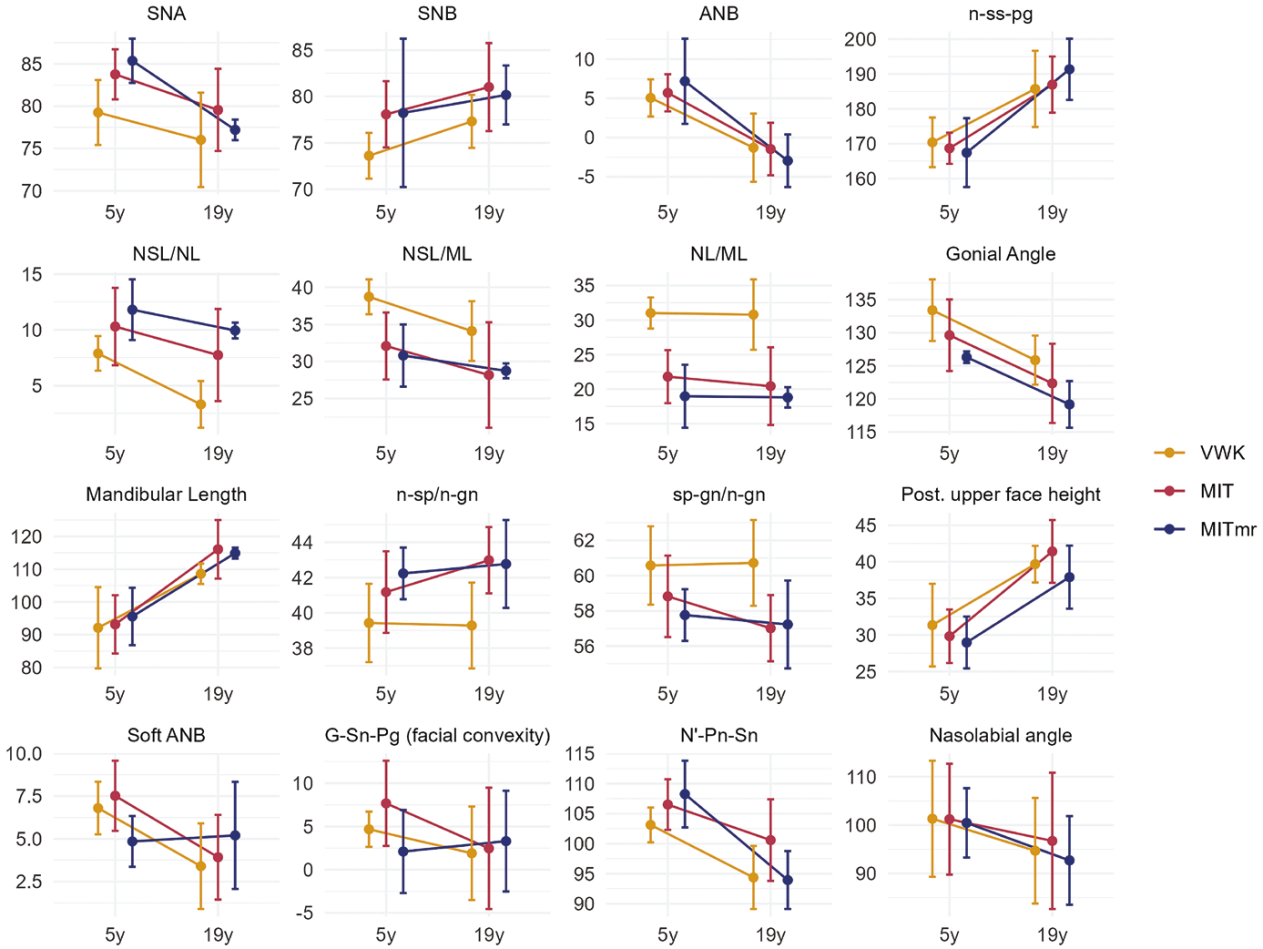
The average value at 5 and 19 years of age with +/− one standard deviation, depending on the surgical techniques (VWK, MIT, MITmr) and age (*n* = 32).

The overall mean intra-examiner reliability varied between 0.1 and 0.69 for angular and between 0.17 and 0.47 for linear measurements (Table 1). There were statistically significant differences between the three surgical techniques (VWK, MIT, and MITmr) for eight of the skeletal variables and for two of the soft-tissue variables at 19 years of age (Table 2). Paired comparisons for each of the variables with a significant overall test from the ANOVA were made and they showed differences between VWK and MIT (VWK-MIT) for the SNB and the nasolabial angle between VWK and MITmr (VWK-MITmr) for posterior upper face height and between both MIT and MITmr compared to VWK (VWK-MIT and VWK-MITmr) for the NSL/NL, NSL/ML, NL/ML, n-sp/n-gn, sp-gn/n-gn, and ILS-NL (Table 3). VWK resulted in a more retrusive maxilla (smaller SNA) than MIT and MITmr. On the contrary, ANB was less negative after VWK than after MIT and MITmr but these differences were not statistically significant. The average values at 19 are shown in Fig. 3. The 32 patients who had lateral cephalograms evaluated at both 5 and 19 years of age are presented separately (Table 4). The average values at both timepoints are shown in Fig. 4. According to the medical charts, 28% of the patients operated with VWK received a pharyngeal flap, compared to 33% operated with MIT but this was not statistically significant (*P *= .68). At the age of 19, 21% of the patients operated with VWK had received orthognathic surgery with an osteotomy to correct the maxillary retrusion, compared to 4.5% of those operated with MIT, and this was a statistically significant difference (*P* = .026).

**Table 1.** The method error, in degrees or millimetres per variable, based on 15 retraced cephalograms (5 at 5 years and 10 at 19 years of age).

	Method error
SNA (°)	0.53
SNB (°)	0.54
ANB (°)	0.42
n-ss-pg (°)	0.69
NSL/NL (°)	0.,51
NSL/ML (°)	0.37
NL/ML (°)	0.32
Gonial Angle (°)	0.48
sp’-pm (mm)	0.49
Mandibular length (mm)	0.32
n-sp’’/n-gn	0.41
sp’’-gn/n-gn	0.41
Post. upper face height (mm)	0.33
Post. face height (mm)	0.27
ILS/NL (°)	0.52
ILI/ML (°)	0.35
ILI/ILS (°)	0.56
Soft ANB (°)	0.1
G-Sn-Pg’ (°)	0.53
Nasolabial angle (°)	0.6
N’-Sn’/N’-Gn’ (mm)	0.37
Upper Lip to E plane (mm)	0.19
Lower Lip to E plane (mm)	0.17

**Table 2.** Cephalometric values at 19 years of age (*n* = 68), divided by surgical techniques (VWK, MIT, and MITmr) presented with mean, standard deviation (SD), and *P*-value.

Surgical technique	VWK	MIT	MITmr	
n	13	39	16	
Variables	Mean (SD)	Mean (SD)	Mean (SD)	*P*- value
SNA (°)	74.50 (3.75)	77.64 (5.30)	76.69 (2.61)	.104
SNB (°)	76.00 (3.82)	79.71 (4.86)	78.75 (3.36)	.036
ANB (°)	−1.50 (3.43)	−2.07 (4.16)	−2.05 (3.07)	.891
n-ss-pg (°)	186.13 (8.51)	188.33 (9.58)	188.11 (8.10)	.761
NSL/NL (°)	4.18 (3.52)	8.23 (3.72)	9.83 (3.04)	<.001
NSL/ML (°)	35.48 (5.00)	28.84 (6.61)	30.28 (4.94)	.006
NL/ML (°)	31.06 (5.00)	20.70 (5.38)	20.44 (5.01)	<.001
Gonial angle (°)	124.58 (4.68)	121.34 (5.66)	121.11 (4.76)	.153
sp´-pm (mm)	49.32 (5.22)	47.36 (3.23)	46.23 (4.56)	.122
Mandibular length (mm)	116.22 (11.38)	114.24 (8.46)	111.61 (7.44)	.381
n-sp/ngn (%)	39.65 (2.56)	43.18 (1.95)	42.88 (2.28)	<.001
sp-gn/n-gn (%)	60.35 (2.56)	56.82 (1.95)	57.12 (2.28)	<.001
Post. upper face height (mm)	43.90 (4.96)	41.24 (3.84)	38.61 (3.47)	.003
Post. face height (mm)	81.62 (9.70)	81.90 (7.49)	78.31 (6.75)	.291
ILS-NL (°)	103.45 (8.55)	113.45 (6.91)	114.87 (8.73)	<.001
ILI-ML (°)	85.50 (6.77)	87.49 (10.32)	84.79 (8.85)	.585
ILI-ILS (°)	139.33 (9.09)	138.02 (11.89)	139.30 (16.64)	.868
Soft ANB (°)	3.42 (2.78)	3.56 (3.32)	3.27 (2.59)	.952
GSnPg`(°)	2.95 (5.57	2.13 (7.87)	1.17 (4.95)	.801
Nasolabial angle (°)	88.56 (9.75)	98.99 (13.93)	96.27 (7.41)	.031
NSnNGn (°)	41.17 (2.36)	44.08 (2.72)	44.08 (2.63)	.006
Upper lip to Eplane (mm)	−6.42 (2.92)	−6.98 (3.11)	−7.68 (3.51)	.587
Lower lip to Eplane (mm)	−0.66 (2.81)	−2.42 (2.97)	−3.06 (3.71)	.129

**Table 3.** Paired comparisons between the surgical techniques (VWK-MIT, MITmr-MIT, and MITmr-VWK) for the variables which showed overall significance with ANOVA.

Surgical technique	VWK-MIT	MITmr-MIT	MITmr-VWK
Variables			
SNB	0.027*	0.743	0.219
NSL/ML	0.004**	0.700	0.067
ML/NL	0.000***	0.984	0.000***
n-sp/n-gn	0.000***	0.885	0.001**
sp-gn/n-gn	0.000***	0.885	0.001**
Post.Upp.Facial Height	0.103	0.075	0.002**
ILS-NL	0.000***	0.809	0.001**
Nasolabial angle	0.024*	0.738	0.218
NSnNGn (soft facial height)	0.006**	1.000	0.020*

^*^ *P*-value < 0.05; **P-value < 0.01; ***P-value < 0.001.

**Table 4.** Cephalometric values at 5 and 19 years of age (*n* = 32), divided by surgical techniques (VWK, MIT, and MITmr) presented with mean and standard deviation (SD).

Surgical technique	VWK:5y	MIT:5y	MITmr:5y	VWK:19y	MIT:19y	MITmr:19y
n	5	24	3	5	24	3
Variables	Mean (SD)	Mean (SD)	Mean (SD)	Mean (SD)	Mean (SD)	Mean (SD)
SNA	79.2 (3.8)	83.8 (3.0)	85.4 (2.6)	76:0 (5.6)	79.6 (4.9)	77.2 (1.2)
SNB	73.6 (2.5)	78.1 (3.6)	78.2 (8.0)	77.3 (2.9)	81.0 (4.7)	80.2 (3.2)
ANB	5.0 (2.4)	5.7 (2.4)	7.2 (5.4)	−1.3 (4.3)	−1.5 (3.3)	−3.0 (3.4)
n-ss-pg	170.4 (7.1)	168.7 (4.5)	167.4 (9.9)	185.7 (10.9)	187.0 (8.1)	191.4 (8.8)
NSL/NL	7.9 (1.6)	10.3 (3.5)	11.8 (2.7)	3.3 (2.1)	7.7 (4.1)	9.9 (0.7)
NSL/ML	38.7 (2.3)	32.1 (4.5)	30.8 (4.2)	34.1 (4.0)	28.2 (7.1)	28.7 (1.0)
NL/ML	31.0 (2.3)	21.8 (3.8)	19.0 (4.6)	30.8 (5.1)	20.4 (5.6)	18.8 (1.5)
Gonial angle	133.4 (4.7)	129.6 (5.4)	126.3 (0.9)	125.9 (3.7)	122.4 (6.0)	119.2 (3.5)
Mandibular length	92.1 (12.4)	93.2 (8.9)	95.6 (8.8)	108.6 (3.1)	116.1 (8.9)	114.9 (1.7)
n-sp/n-gn	39.4 (2.2)	41.2 (2.3)	42.2 (1.5)	39.3 (2.4)	43.0 (1.9)	42.8 (2.5)
sp-gn/n-gn	60.6 (2.2)	58.8 (2.3)	57.8 (1.5)	60.7 (2.4)	57.0 (1.9)	57.2 (2.5)
Post. upper face height	31.3 (5.7)	29.8 (3.7)	29.0 (3.5)	39.7 (2.5)	41.4 (4.3)	37.9 (4.3)
Soft ANB	6.8 (1.5)	7.5 (2.1)	4.8 (1.5)	3.4 (2.5)	3.9 (2.5)	5.2 (3.1)
G-Sn-Pg´(facial convexity)	4.7 (2.0)	7.7 (4.9)	2.1 (4.8)	1.9 (5.4)	2.5 (7.0)	3.3 (5.8)
N´-Pn-Sn	103.1 (2.9)	106.5 (4.2)	108.3 (5.6)	94.4 (5.3)	100.6 (6.8)	93.9 (4.8)
Nasolabial angle	101.3 (12.0)	101.2 (11.5)	100.5 (7.2)	94.7 (10.9)	96.7 (14.1)	92.7 (9.2)
Age	5.0 (0.7)	5.0 (0.0)	5.0 (0.0)	18.6 (0.5)	19.2 (0.6)	19.0 (0.0)

### Comparison between long-term outcomes of VWK, MIT, and MITmr and normative data as well as outcomes from other centres

All three surgical techniques resulted in significantly less favourable cephalometric values, to varying degrees, compared to normative values for most cephalometric variables except for ILI-ILS (Table 5). The SNA angle was less obtuse than normative data for both VWK, MIT, and MITmr, and SNB was significantly less obtuse only for VWK. The ANB angle was significantly less favourable for all three techniques but to a greater degree after MIT and MITmr. VWK did not differ from normative values regarding NSL/NL, n-ss-pg, and ILS-NL, but for NSL/ML, the difference was significant as well as for NL/ML. Conversely, MIT and MITmr differed significantly from the norm regarding NSL/NL, n-ss-pg, and ILS-NL but not for NSL/ML and NL/ML.

**Table 5.** Comparing norm values to each of the three surgical techniques. All tests are independent *t*-test based on the given *n*, mean and standard deviation. Adjusted *P*-values are Bonferroni adjusted *P*-values for *m* = 3 tests within each outcome variable.

		Norm.data			This study									
Variable	Technique	*n*	Mean	SD	*n*	Mean	SD	*T*-value	Estimate	CI 95% lower	CI 95% upper	*P*-value	Adjusted *P*-value	Signif
SNA	MIT	33	83.0	3.4	39	77.6	5.3	5.22	5.4	3.33	7.47	.000	.000	***
SNA	MITmr	33	83.0	3.4	16	76.7	2.6	7.17	6.3	4.52	8.08	.000	.000	***
SNA	VWK	33	83.0	3.4	13	74.5	3.8	7.10	8.5	6.01	10.99	.000	.000	***
SNB	MIT	33	81.1	3.3	39	79.7	4.9	1.44	1.4	−0.54	3.34	.155	.464	
SNB	MITmr	33	81.1	3.3	16	78.8	3.4	2.24	2.3	0.20	4.40	.033	.098	
SNB	VWK	33	81.1	3.3	13	76.0	3.8	4.25	5.1	2.59	7.61	.000	.001	**
ANB	MIT	33	1.8	2.1	39	−2.1	4.2	5.09	3.9	2.37	5.43	.000	.000	***
ANB	MITmr	33	1.8	2.1	16	−2.1	3.1	4.55	3.9	2.12	5.68	.000	.000	***
ANB	VWK	33	1.8	2.1	13	−1.5	3.4	3.26	3.3	1.15	5.45	.005	.015	*
NSL/NL	MIT	33	6.3	2.6	39	8.2	3.7	−2.55	−1.9	−3.39	−0.41	.013	.039	*
NSL/NL	MITmr	33	6.3	2.6	16	9.8	3.0	−4.00	−3.5	−5.30	−1.70	.000	.001	**
NSL/NL	VWK	33	6.3	2.6	13	4.2	3.5	1.96	2.1	−0.16	4.36	.066	.198	
NSL/ML	MIT	33	28.5	4.4	39	28.8	6.6	−0.23	−0.3	−2.91	2.31	.819	1.000	
NSL/ML	MITmr	33	28.5	4.4	16	30.3	5.0	−1.23	−1.8	−4.81	1.21	.230	.691	
NSL/ML	VWK	33	28.5	4.4	13	35.5	5.0	−4.42	−7.0	−10.31	−3.69	.000	.001	***
NL/ML	MIT	33	21.7	5.0	39	20.7	5.4	0.82	1.0	−1.45	3.45	.418	1.000	
NL/ML	MITmr	33	21.7	5.0	16	20.4	5.0	0.85	1.3	−1.81	4.41	.400	1.000	
NL/ML	VWK	33	21.7	5.0	13	31.1	5.0	−5.74	−9.4	−12.80	−6.00	.000	.000	***
*n*-ss-pg	MIT	33	179.4	4.5	39	188.3	9.6	−5.16	−8.9	−12.36	−5.44	.000	.000	***
*n*-ss-pg	MITmr	33	179.4	4.5	16	188.1	8.1	−4.01	−8.7	−13.23	−4.17	.001	.002	**
*n*-ss-pg	VWK	33	179.4	4.5	13	186.1	8.5	−2.70	−6.7	−12.00	−1.40	.017	.050	
*N*-ANS’/ *N*-G	MIT	33	44.7	2.8	39	43.2	2.0	2.59	1.5	0.34	2.66	.012	.037	*
*N*-ANS’/ *N*-G	MITmr	33	44.7	2.8	16	42.9	2.3	2.39	1.8	0.27	3.33	.022	.067	
*N*-ANS’/ *N*-G	VWK	33	44.7	2.8	13	39.7	2.6	5.74	5.0	3.20	6.80	.000	.000	***
ILI/ILS	MIT	33	133.7	8.2	39	138.0	11.9	−1.81	−4.3	−9.05	0.45	.075	.226	
ILI/ILS	MITmr	33	133.7	8.2	16	139.9	16.6	−1.41	−6.2	−15.40	3.00	.174	.523	
ILI/ILS	VWK	33	133.7	8.2	13	139.3	9.1	−1.93	−5.6	−11.65	0.45	.068	.203	
ILS/NL	MIT	33	108.6	6.3	39	113.5	7.0	−3.12	−4.9	−8.03	−1.77	.003	.008	**
ILS/NL	MITmr	33	108.6	6.3	16	114.9	8.7	−2.59	−6.3	−11.34	−1.26	.017	.050	*
ILS/NL	VWK	33	108.6	6.3	13	103.5	8.6	1.94	5.1	−0.43	10.63	.068	.205	

^*^ *P*-value < 0.05; **P-value < 0.01; ***P-value < 0.001.

The cephalometric long-term outcome, at terminated mid-facial growth, from other cleft-centres’ previously reported results is presented in Table 6.

**Table 6.** Comparison between the cephalometric long-term outcome in the present study, normative data, and results from other cleft centres.

	Thilander *et al.*, 2005	VWK	MIT	MITmr	Gaggl *et al.*, 2003	Brattstrom *et al.*, 2005	Farzaneh *et al.*, 2008	Friede *et al.*, 2012	Jabbari *et al.*, 2017	Nollet *et al.*, 2008	Kappen *et al.*, 2017
Town, Country	Göteborg, Sweden	Stockholm, Sweden	Stockholm, Sweden	Stockholm, Sweden	Graz, Austria	Eurocleft, Center A	Malmö, Sweden	Göteborg, Sweden	Uppsala, Sweden	Nijmegen, the Netherlands	Utrecht, the Netherlands
Type of palatal repair	normal pop., pooled f+m	one-stage+ early bg	one-stage	one-stage	one-stage	two-stage	one-stage (27W+34vL)	two-stage	two-stage	two-stage	two-stage
Timing of repair		19 m	13 m	13 m	11–14 m	s.p. 9–18 m	W 18 m	s.p. 3 m	s.p. 6 m	s.p. 12–14 m	s.p. 8 m
						h.p. 8–11y	vL 8 m	h.p. 8 y	h.p. 1–3 y	h.p. 9–11 y	h.p. 3 y
N of patients	33	13	39	16	30	24	61	50	29	37	52
Mean age (SD)	19	18.9 (0.7)	19.1 (0.9)	19.1 (0.1)	18.4	17	>20	18.9 (0.4)	18	18 (1.2)	21 (3.4)
Variables sagittal	M (SD)	M (SD)	M (SD)	M (SD)	M (SD)	M (SD)	M (SD)	M (SD)	M (extrapol. from graph)	M (SD)	M (SD)
SNA	83.0 (3.4)	74.5 (3.75)	77.6 (5.3)	76.7 (2.6)	78.2 (0.6)	74.5 (4.4)	76.5 (4.1)	78.8 (3.2)	82.7	74.3 (4.5)	74.9 (4.19)
SNB	81.1 (3.3)	76.0 (3.8)	79.7 (4.9)	78.8 (3.4)	76.8 (0.5)		78.7 (4.3) m, 74.7 (4.2) f	76.8 (2.6)	80.3		75.7 (3.73)
ANB	1.8 (2.1)	−1.5 (3.4)	−2.1 (4.2)	−2.1 (3.1)	1.8 (0.5)	−0.1 (2.5)	−1.6(1.9) m, 1.1 (1.4) f	−0.1 (2.5)	2.1	−0.4 (3.8)	−0.9 (2.71)
*n*-ss-pg	179.4 (4.5)	186.1 (8.5)	188.3 (9.6)	188.1 (8.1)				182.6 (6.1)			
Variables vertical	M (SD)	M (SD)	M (SD)	M (SD)	M (SD)	M (SD)	M (SD)	M (SD)	M (extrapol. from graph)	M (SD)	M (SD)
NSL/NL	6.3 (2.6)	4.2 (3.5)	8.2 (3.7)	9.8 (3.0)	9.3 (0.4)	8.9 (4.1)	8.8 (4.1) m, 12.4 (4.1) f	8.8 (3.2)	3.9	9.5 (3.6)	8.5 (3.90)
NSL/ML	28.5 (4.4)	35.5 (5.0)	28.8 (6.6)	30.3 (5.0)	36.4 (0.7)	37.2 (5.9)	32.4 (8.1)	32.8 (5.8)	30.3	35.7 (6.9)	35.4 (6.35)
NL/ML	21.7 (5.0)	31.1 (5.0)	20.7 (5.4)	20.4 (5.0)	26.7 (0.7)		22.1 (7.7)	24.0 (5.1)	30.3		26.5 (5.84)
N-Ans/N-Gn × 100 (*:*N*-ANS’/ *N*-G)	44.7 (2.8)	39.7 (2.6) *	43.2 (1.95) *	42.9 (2.3) *		42.1 (2.2)	38.8 (7.5) m, 39.1 (6.1) f	42.7 (1.7)		44.1 (2.0)	41.0 (2.5)
Dento alveolar	M (SD)	M (SD)	M (SD)	M (SD)	M (SD)	M (SD)	M (SD)	M (SD)	M (extrapol. from graph)	M (SD)	M (SD)
ILS/ILI	133.7 (8.2)	139.3 (9.1)	138.0 (11.9)	139.9 (16.6)		127.8 (13.4)				131.7 (12.1)	128.2 (8.50)
ILS/NL	108.6 (6.3)	103.5 (8.6)	113.5 (7.0)	114.9 (8.7)		95.8 (5.8)	112.1 (7.5)	103.0 (6.1)		111.0 (6.3)	110.2 (6.98)

### Predictive analysis

The overall relation between baseline values at 5 years and outcome at 19 years is strong, whereas surgical technique had limited influence. The variability in the outcome explained by the given models (*r*^2^) varied between 18% and 52%. (Table 7). The only outcome at 19 years that continued to present a statistically significant relation to surgical technique (when adjusted for baseline values at 5 years) was face height.

**Table 7.** Linear regression analysis of cephalometric values at 19 years of age.

	Estimate	DF	*P* value-value at 5 y	*P*-value-surg.technique	*r* ^2^
SNA	1.011	27	.000	.219	0.433
SNB	0.671	28	.000	.871	0.410
SNPg	0.730	28	.000	.875	0.412
ANB	0.563	27	.031	.330	0.217
*n*-ss-pg	0.798	27	.010	.290	0.276
NSL/NL	0.157	27	.138	.058	0.243
NSL/ML	0.774	28	.001	.887	0.335
NL/ML	0.379	27	.000	.086	0.447
Gonial angle	0.748	28	.000	.909	0.519
Mandibular length	0.517	28	.000	.111	0.437
*n*-sp/*n*-gn	−0.029	27	.292	.001	0.437
sp-gn/*n*-gn	−0.029	27	.292	.001	0.437
Post. upper face height	0.634	28	.000	.131	0.429
Soft ANB	0.823	25	.000	.561	0.420
GSnPg (facial convexity)	0.892	25	.001	.709	0.370
Nasolabial angle	0.493	26	.029	.856	0.179

## Discussion

### Comparison between VWK, MIT, and MITmr and between the three surgical techniques and normative data

The width of the cleft palate was not recorded for the subjects in the present study and hence the differences in outcome could depend not only on the type of surgical technique used but also on the characteristics of the initial cleft. Some authors have shown that there is a significant negative relationship between cleft width on infant casts and maxillary growth [22, 23]. However, Russell *et al.* [24] showed that infant cleft size does not affect the eventual dental arch relationship in the mixed dentition in patients with complete UCLP. In most other studies comparing cephalometric outcomes the initial cleft width is not accounted for. In 1987, the treatment protocol in Stockholm was changed in two ways simultaneously. The technique for palatal repair was changed from VWK to MIT at the same time as the timing of bone grafting of the cleft alveolus was changed from early (at the time of the lip- and nose plasty) to bone grafting in the mixed dentition. It has already been shown that early bone grafting inhibits maxillary growth, and therefore, it is not clear if it is this parameter, or the technique of the palatal surgery that is responsible for the poorer results in the VWK group. Yet, it is known that VWK seems to have a more negative impact on midface development, mainly in the transverse dimension [14], compared to MIT also in patients with isolated cleft palate, where no alveolar bone grafting was performed at all. It also remains unclear if preoperative maxillofacial orthopaedics (T-traction) produces a more negative effect on dental arch relationships than a palatal plate. However, a previous study suggests that a more normal anatomy of the cleft region occurs during the first 6 months of life whether T-traction is used or not [25]. Similarly, infant orthopaedic treatment could not be found to have an impact on the dental arch relationships in the deciduous dentition according to Bongaarts *et al.* [26]. Patients operated with all three types of one-stage palatal repair in the cohort had a more retrusive maxilla (SNA) and greater maxillary retrusion in relation to the mandible (ANB) compared to individuals born without a cleft. The negative relationship between the maxilla and the mandible (ANB) was greater in the MIT and MITmr groups compared to VWK, even though the difference was not statistically significant. This might in part be due to a significantly less pronounced mandible (lower SNB value) in the VWK group compared to the MIT and MITmr groups (which had normal values). On the other hand, the proclination of the upper incisors (ILS-NSL), which is likely caused by orthodontic treatment to compensate for the position of the underlaying jaw, was significantly greater in the MIT and MITmr groups compared to the VWK group (which showed even less proclination than the reference group). However, this in turn might be explained by the fact that orthodontic treatment, especially regarding treatment with fixed appliances, was less available to the patients in the VWK group (born 1975–1986). The MIT and MITmr techniques resulted in a more anterior inclination of the maxilla (NSL/NL), while the VWK technique resulted in a more posterior inclination of the maxilla compared to the norm. The MIT and MITmr techniques resulted in a significantly flatter facial profile (n-ss-pg) compared to that of individuals born without a cleft. The total face height was greater than the reference values in all three study groups. The anterior upper face height (n-sp/n-gn) was significantly lower in the VWK group compared to the MIT and MITmr groups and compared to the norm. This could also be seen for the corresponding soft tissue variables. The vertical relations between the maxilla and mandible (NL-ML) were more open in the VWK group compared to the MIT and MITmr groups (which presented normal values). The vertical relation of the mandible in relation to the skull base (NSL/ML) was significantly more obtuse in the VWK group than in the other groups (MIT and MITmr), which had an angulation like that of reference values. However, the posterior upper face height (pm’-pm) was significantly higher in the VWK group than in the MIT and MITmr groups. Considering the soft tissue variables, significant differences were seen between MIT, MITmr, and VWK where the two latter ones resulted in a less pointy nose (lower N’-Pn-Sn angle) and hence a greater nasolabial angle was found in the MIT group.

### Comparison of the results of the present cohort and previous long-term outcomes of other one-stage palatal repair techniques

In a study by Farzaneh *et al.* [27] 34 adult patients were operated at 8 months with von Langenbeck technique (L-8), and 27 patients were operated at 18 months according to the Wardill technique (W-18) no differences were found between the groups, only between genders. In fact, in both groups from Malmö the females had ANB values closer to the reference group and in the males a degree of maxillary retrusion like that of the patients in the VWK group in the present study was seen. Most cephalometric variables were similar between L-8, W-18, and MIT/MITmr. However, the relation of the palatal plane to the mandibular plane (NL/ML) was greater in the VWK group, and the maxillary inclination (NSL/ML) was lower compared to L-8 and W-18, MIT/MITmr, and the reference values. Despite the similar operation technique and timing in the W-18 and the VWK groups, there was one important difference namely that the patients in the latter group were bone-grafted at the same time as the lip repair was performed. Early bone grafting to the alveolar cleft has been shown to have a deleterious effect on maxillary growth [28–30] and could hence explain the less favourable outcome in the VWK group compared to the W-18 group.

In the study by Parikakis *et al.* [31] where patients born with isolated cleft palate were operated with MIT or MITmr by the same surgeons as in the present patient cohort and where the tracer, of cephalograms at 16 years of age, was the same (Costas Parikakis) the results showed both retrognathic maxillas and mandibles. Smaller antero-posteriorly and posteriorly inclined maxillas were also found in the treated children compared to a group of patients born without cleft. Cephalometric variables of patients born with isolated cleft palate and operated at our centre with VWK and MIT, respectively, were compared at 5 and 10 years of age by Parikakis *et al.* [32]. Only minor differences in cephalometric morphology were found between the groups which might imply either that the difference between different surgical techniques only can be detected at an older age and/or that the surgery of the lip and/or alveolar cleft produces a difference in growth in addition to the palatal repair. However, when dental arch relationships were measured, according to the GOSLON Yardstick, in the same patient cohort, the VWK technique resulted in a greater degree of maxillary retrusion than MIT, and the difference was statistically significant at 19 years of age [33].

Two study groups from Graz, where patients in the first group had been operated with a one-stage palatoplasty technique (according to Veau and hence similar to the VWK group in the present study) at 11 to 14 months of age were compared by Gaggl *et al.* [34]. At a mean of 18.4 years, the patients in the Veau group presented with cephalometric values similar to those of the VWK group in the present study, except for SNA and NSL/NL which were more favourable than after VWK and similar to the values of the MIT/MITmr groups. In the Veau study group from Graz, no maxillary retrusion (i.e. normal ANB) after one-stage repair was observed. However, the second study group where patients had been operated with a two-stage technique an impairment of growth of the maxilla in the sagittal plane was seen. In that group, the soft palate was closed at 18–24 months and the hard palate at 6 years of age with Veau palatal pedicle flaps. Hence, the technique of soft palate repair in the study by Gaggl *et al.* showed a negative effect on the maxillary growth which is contradictory as delayed closure of the hard palate often is reported to produce better facial growth [35–37]. However, in the systematic review by Liao *et al.* [38] it was concluded that the effect of the timing of hard palate repair on facial growth could not be evaluated due to methodological flaws and the heterogeneity of study groups.

### Comparison between the results of the present cohort and previous long-term outcomes of two-stage palatal repair techniques

Long-term results from centres where different types of two-stage techniques for palatal repair were evaluated in adult patients born with UCLP [39–41] showed similar SNA, NSL/NL, and SNB values to those after MIT/MITmr in the present study. The total face angulation (NSL/ML) was more obtuse after two-stage techniques compared to MIT, which presented an almost normal value. The relation of the palatal plane to the mandibular plane (NL/ML) was also closer to normal values in the MIT and MITmr groups than in VWK and all other centres where the mandibular plane was more posteriorly inclined. However, the maxillary retrusion in relation to the mandible was significantly greater (negative ANB) in these groups compared to all others, including VWK, which seems contradictory. As Kappen *et al.* [41] noted, there was a trend towards worse ANB values in centres with early palate closure in the Eurocleft study [4] but the difference in timing between VWK and MIT/MITmr was only 3 months. Hence, the timing of surgery is probably not the only factor influencing mid-facial growth. The inter-incisor angle (ILS-ILI) was greater than reference data in all three study groups from Stockholm and lower in the two-stage study groups, suggesting a result of orthodontic treatment with an over-proclination of the upper incisors in the latter ones. The study by Jabbari *et al.* [42], with a two-stage repair, presented the best cephalometric outcome of all centres except for NL/ML, showing a mandibular plane significantly more posteriorly inclined compared both to MIT/MITmr and the one-stage groups from Malmö [27].

Meazzini *et al.* [43] compared short- and long-term craniofacial growth after two different types of two-stage protocols for palatal repair. This study was not included in Table 4 since the cephalograms of patients in the Milan protocol and the Oslo protocol were analysed at a mean of 16.5 and 16.8 years, respectively. No significant cephalometric difference in the maxillary prominence at 5 years, a mild but significant difference at 10 years, and again no difference at the older age were found. Nevertheless, the need for orthognathic surgery was larger in the Milan sample (26%) than in the Oslo sample (13%). In the study by Bakri *et al.* [44] the Wardill–Kilner (W-K) protocol was compared to the Gothenburg delayed hard palate closure (DHPC) protocol in patients with complete UCLP at 10 years of age. They found a more normal anterior maxillary vertical growth and overbite and therefore increased maxillary inclination after the two-stage protocol. Similarly, Fudalej *et al.* [45] compared cephalometric outcome between the Oslo two-stage protocol (with early hard palate closure) and the Warsaw one-stage protocol in 61 patients at a mean age of 10.9 years and concluded a more favourable outcome in the Oslo group. However, it is unclear if the results in these two latter studies remained the same after terminated facial growth.

The craniofacial growth and morphology in children born with UCLP at 8 years of age in the Scandcleft study [46], a large multicenter randomized controlled trial, showed significant maxillary retrusion as well as reduced intermaxillary relationships compared to age-matched children without a cleft. The results from the different study arms showed some significant differences but since they were not major, the results were pooled for SNA and SNB angles. The upper and lower incisors were retroclined, and the vertical jaw relationship was decreased. The authors expect these differences to deteriorate as the children grow older, and it is possible that some differences between the study arms will be more apparent with age.

### Comparison between cephalometric outcome at 5 and 19 years of age-predictive analysis

Regarding predictive data on mid-facial long-term growth, there were 32 patients who had lateral cephalograms at both 5 and 19 years of age and hence the groups divided by surgical technique were very uneven and small (VWK, *n* = 5 and MITmr, *n* = 3). When comparing the patients who did not have a cephalogram at 5 years (*n* = 36) to the complete group, it was found that these patients had significantly lower values for SNA, SNB, and SNPg. This difference remained when the MIT group (which was the largest) was examined separately. It is unclear what this difference stands for. Only 18–52% of the variance at 19 could be explained by the outcome at 5 years of age, when the set of data was adjusted for surgical technique. Hence, for all other cephalometric variables, most of the variance was not explained by the model, but this could depend on the small sample (*n* = 32). Only the face height (s-n-pg) and the gonial angle showed a high correlation between the values at 5 years and the values at 19 years of age. These cephalometric variables are not considered as being directly influenced by primary surgery, but one hypothesis could be that the growth of the gonial angle is influenced by a retrusive maxilla. However, it is known that the inclination of the lower border of the mandible is almost the same in the adult as in the child [47]. There are some previous studies in contrast with the results in the present investigation. In fact, Nollet *et al.* [40] showed that for most of the cephalometric values at age 18, 40–80% of the variance could be explained by the cephalometric values at a younger age. Several cephalometric variables at age 9 (ANB, s-n-pg, and the corresponding soft tissue variables) were significant predictors for the need for surgery at 18 years of age. However, it seemed that it was more difficult to predict the cephalometric outcome at 18 years if the patient had unfavourable results at 8 years of age. The need for orthognathic correction at age 18 was correctly predicted from age 9 for 85% of the investigated patient group. Similarly, Yun-Chia Ku *et al.* [48] found that at 11 years of age, the variables ANB, overjet, and maxillary retrusion could predict the future need for orthognathic surgery in patients born with UCLP at a mean age of 18.5 years, with an accuracy of almost 90%. Lin *et al.* [49] evaluated lateral cephalograms in patients born with UCLP from age six and to at least 15 years of age (mean 16.7 years). They concluded that it was possible to predict, using machine learning, the need of future orthognathic surgery at the age of 6 years. However, other authors believe that it is impossible to predict a need for later maxillary osteotomy during the transitory dentition [50].

Since making a comparison between the mean values of all three groups of patients in the present study with those of previously published studies, with independent tests, would imply a high risk of making type 1 error this analysis was not performed.

The first null hypothesis was rejected since statistically significant differences could be found between the three techniques, especially between VWK and MIT/MITmr, even though these differences were not major. The second null hypothesis was partly rejected because there seems to be a relation between facial growth at 5 and 19 years of age for some cephalometric variables.

## Limitations

This is a retrospective study with some limitations. One limitation is that as many as five surgeons operated the patients in the cohort. However, considering that this was over a period of almost 30 years, it is quite normal for a large cleft centre to have more dedicated surgeons. Another limitation is that hypodontia, which is present outside the cleft region in 15.5% of individuals with different types of CLP [51], was not recorded in this study although it is known that the absence of the permanent lateral incisor has a negative effect upon maxillary protrusion [52–54]. The cleft lateral was missing in 43.8% of the 425 individuals in a study by Rizell *et al.* [55] and a statistically significant difference was found for ANB between individuals with agenesis of two or more maxillary teeth [56]. In future studies, it would be interesting to evaluate if this difference is still evident at the end of skeletal maturation.

The uneven spread of patients in the separate treatment groups, with more patients operated with the MIT technique than with VWK or MITmr, might also be considered as problematic. In addition, there were more patients with cephalograms at 5 and 19 years of age who had been operated with MIT compared to VWK and MITmr. However, since it is a retrospective study and it was both impossible to retrieve older material and not reasonable to wait even longer with the evaluation to include more patients in the MITmr group, it was seen as an inherent but acceptable setback of the study design.

An important limitation of this study is missing data. Patient records were retrieved over a long period of time, including x-rays, and filing systems have been changed from analogue to digital programs. Families often moved before the follow-up was terminated and some were operated with different techniques. In the present long-term study, all lateral cephalograms of adolescents younger than 17 years of age were excluded to examine patients only at the end of skeletal maturation.

The greatest concerns are that it remains unknown if the patients who have measurements at 5 years systematically differ from patients who do not and that the study might be underpowered.

## Conclusion

Studies reporting on long-term follow-up and outcomes after multidisciplinary treatment of UCLP are imperative to determine if some surgical techniques for cleft repair are preferable to others. MIT and MITmr resulted in better cephalometric results regarding facial growth sagittally and vertically compared to VWK, even though the differences were not major. It seems that some variables in cephalometric analysis at 5 years of age, especially regarding face height, might predict the outcome at 19 years of age. However, a larger cohort of patients would be needed to make definitive conclusions.

## Data Availability

The datasets analysed during the current study are available from the corresponding author on reasonable request.

## Notes

A part of this manuscript was orally presented at the 14th International Cleft Congress, 11–15 of July 2022, in Edinburgh, UK.
